# A Comparative Study of Two *In Vivo* PET Verification Methods in Clinical Cases

**DOI:** 10.3389/fonc.2021.617787

**Published:** 2021-09-03

**Authors:** Junyu Zhang, Yan Lu, Yinxiangzi Sheng, Weiwei Wang, Zhengshan Hong, Yun Sun, Rong Zhou, Jingyi Cheng

**Affiliations:** ^1^ Department of Nuclear Medicine, Shanghai Proton and Heavy Ion Center, Fudan University Cancer Hospital, Shanghai, China; ^2^ Shanghai Key Laboratory of Radiation Oncology, Shanghai, China; ^3^ Shanghai Engineering Research Center of Proton and Heavy Ion Radiation Therapy, Shanghai, China; ^4^ College of Physics, Sichuan University, Chengdu, China; ^5^ Key Laboratory of Radiation Physics and Technology, Ministry of Education, Chengdu, China; ^6^ Department of Medical Physics, Shanghai Proton and Heavy Ion Center, Shanghai, China; ^7^ Department of Radiotherapy, Shanghai Proton and Heavy Ion Center, Shanghai, China

**Keywords:** proton therapy, breast cancer, positron emission tomography, depth verification, methods comparison

## Abstract

**Purpose:**

Positron emission tomography (PET) range verification is an important method that can help improve the confidence in proton therapy for clinical applications. Two kinds of verification methods are implemented and compared based on clinical cases in this study.

**Method:**

The study is conducted on 14 breast cancer patients following proton irradiation treatment. Verification is done by calculating the depth error between the numerically predicted values with the measured PET image along the beam direction. Point-based and segment-based methods are applied and compared. The verification results are presented as depth error means and standard deviations in a region of interest (ROI).

**Results:**

The mean value of the depth error of all 14 cases is within the range of [−3, 3] mm for both point-based and segment-based methods, and only one case result calculated by the point-based method is slightly beyond −3 mm. When comparing the mean depth error from the two methods, the paired *t*-test result shows that the *p*-value is 0.541, and the standard deviation of the segment-based method is smaller than that of the point-based method.

**Conclusion:**

In breast cancer case verification application, point-based and segment-based methods show no significant difference in the mean value of results. Both methods can quantify the accuracy of proton radiotherapy to the millimeter level.

## Introduction

A proton beam demonstrates a good dose distribution with a clear edge because of the presence of the Bragg peak on the dose depth deposition curve. Proton therapy is widely utilized to treat solid tumors close to critical organs, as the clear dose edge is good at sparing normal tissue while destroying tumor tissue. The range verification methods, which are necessary to check the irradiation accuracy, are applied to patients during or/and after proton therapy to ensure the proton beam delivered the dose to tumor tissue precisely.

Many *in vivo* non-invasive verification methods are developed (1); positron emission tomography (PET) is one of the most widely used verification techniques following proton irradiation. Positron emitter isotopes are generated by proton beam decay and release a positron. The positron annihilates with an electron and releases a pair of annihilation photons, which is recorded by the coincident detecting system. Using a suitable reconstruction algorithm, the positron distribution image can be reconstructed to reveal quantified information about beam irradiation.

Proton therapy PET verification has been developed through many phantom-based studies (2–5) and clinical cases (6–9). PET verification is assessed by comparing the measured PET image with the predicted PET image using two widely employed methods (3, 10): point-based and segment-based. The point-based method determines the PET depth by a marked point on the PET activity curve along the beam direction, while the segment-based method determines the depth difference relying on a segment of the curve. Several published articles report that the point-based method has a lower robustness against noise and other fluctuations (11, 12), whereas the segment-based method provides higher robustness to curve fluctuation (3, 10). However, these reports have few descriptions on the implementation of the segment-based method, and the comparison between these two methods is not described thoroughly.

Here, we are going to evaluate point-based and segment-based verification methods and investigate the difference between the two methods in clinical application. Both methods are implemented and employed to verify PET depth range. The comparison is performed on breast cancer cases of PET examination following proton therapy.

## Materials and Methods

### Patient Set and PET Prediction

This retrospective study was approved by the ethics committee of Shanghai Proton and Heavy Ion Center, and the requirement for informed consent was waived. In this work, 14 breast cancer patients were analyzed, whose information is listed in **Table 1**. After proton beam treatment, patients underwent a PET scanning *via* the nuclear medicine PET device. All patients have two irradiation fields. In the treatment plans, fractional dose on the clinical target volume (CTV) was 2.0 or 2.66 Gray of photon Equivalent dose (GyE) in different cases, with a boost area (if exists) 20% higher than the CTV area. The relative biological effect (RBE) of proton beam is a fixed value 1.1.

**Table 1:** Patient Information.

Case No.	Age (years)	Dose prescription/(GyE/fx)		Dose distribution	Fields	Time course/min		Δ*R*_50_ - Field 1/mm		Δ*R*_50_ - Field 2/mm		Δ*R_shift_* - Field 1/mm		Δ*R_shift_* - Field 2/mm	
		CTV	Boost			Delay	Acq	CTV	Boost	CTV	Boost	CTV	Boost	CTV	Boost
1	41	2.66	–	Uniform	2	6	20	−0.51 ± 2.51	–	−0.51 ± 2.35	–	−0.90 ± 2.09	–	−1.05 ± 1.99	–
2	50	2.66	–	Uniform	2	13	20	−1.46 ± 3.05	–	−1.52 ± 3.10	–	−1.61 ± 2.28	–	−1.69 ± 2.33	–
3	74	2.00	–	Uniform	2	15	20	0.73 ± 2.45	–	0.69 ± 2.34	–	0.60 ± 1.82	–	0.59 ± 1.78	–
4	35	2.66	–	Uniform	2	13	20	0.78 ± 2.55	–	0.78 ± 2.65	–	0.70 ± 2.04	–	0.73 ± 2.32	–
5	44	2.00	–	Uniform	2	13	20	−2.63 ± 4.71	–	−2.53 ± 4.43	–	−2.59 ± 2.75	–	−2.66 ± 2.84	–
6	61	2.66	–	Uniform	2	9	20	2.18 ± 3.79	–	2.16 ± 3.72	–	2.25 ± 4.67	–	2.13 ± 4.12	–
7	48	–	2.50	boost	2	7	20	–	−1.41 ± 2.64	–	−1.45 ± 2.36	–	−1.88 ± 1.86	–	−1.91 ± 1.71
8	48	–	2.00	boost	2	14	20	–	1.81 ± 1.87	–	1.32 ± 1.74	–	1.42 ± 1.52	–	1.29 ± 1.52
9	39	2.67	3.20	SIB	2	7	20	−3.43 ± 3.36	−2.34 ± 3.83	−3.44 ± 3.56	−2.50 ± 4.34	−2.79 ± 2.08	−1.78 ± 1.52	−2.72 ± 2.03	−1.97 ± 1.67
10	63	2.00	2.40	SIB	2	9	20	−1.16 ± 3.24	1.48 ± 2.32	−1.14 ± 3.29	1.54 ± 2.11	−1.28 ± 2.65	0.95 ± 1.57	−1.22 ± 2.66	0.93 ± 1.36
11	49	2.67	3.20	SIB	2	8	20	−2.35 ± 3.12	−2.38 ± 2.04	−2.50 ± 3.16	−2.41 ± 1.99	−1.86 ± 2.21	−1.52 ± 1.36	−1.86 ± 2.16	−1.54 ± 1.25
12	50	2.67	3.20	SIB	2	9	20	0.45 ± 2.79	0.45 ± 2.79	0.60 ± 2.19	0.94 ± 2.40	0.66 ± 1.69	1.24 ± 1.45	0.70 ± 1.72	1.37 ± 1.55
13	42	2.67	3.20	SIB	2	11	20	−0.99 ± 4.42	−0.75 ± 2.22	0.26 ± 5.39	−0.23 ± 1.60	−1.14 ± 1.81	−0.04 ± 1.30	−1.94 ± 2.89	0.09 ± 0.76
14	33	2.67	3.20	SIB	2	8	20	−1.20 ± 2.91	1.68 ± 2.00	1.10 ± 6.24	−2.38 ± 3.00	−1.78 ± 1.82	−2.67 ± 1.95	0.69 ± 3.25	−1.71 ± 2.60

Given patient computer tomography (CT) data and treatment plan, the predicted PET image can be calculated by an analytical method (13). A group in Ludwig-Maximillian Universität (LMU) has developed a PET prediction module based on the Treatment Planning System (TPS) working frame (14), which calculates the predicted PET by convolving the dose with a filter function spot by spot and finally generates the PET distribution with washout (10, 15). This module is applied in this work as the source of predicted PET data.

### PET Acquisition and Data Preprocess

In this work, the patient PET data are acquired by “offline” PET, which means that patients after proton irradiation will be transferred over a distance to the PET device; thus, the signal intensity decreased before the acquisition. Patients needed around 5–10 min to transport from the treatment room to the PET room. PET images are acquired by Biograph mCT (16) (Siemens), a PET/CT coupled device that acquires PET and CT data under the same geometry frame. It took us 20 min to acquire patient data. Furthermore, we reconstructed the images with ordered subsets expectation maximization algorithm (17, 18). In addition, CT attenuation correction was applied to reduce the signal attenuation caused by self-absorption. Since the predicted PET follows patient plan CT frame and measured PET follows the PET-CT frame, the plan CT is rigidly co-registered to the PET-CT and thus predicted and measured PET can be compared under one geometry frame.

Because of the low intensity of the PET signal, the reconstructed PET image has a lot of ripples, and the image has lost the accurate PET activity information; thus, measured PET is not suitable for a direct comparison with the predicted PET. A simple moving-average smoothing was applied to the original measured image to smoothen the ripples and then normalized to the scale of the predicted image (19). It is now possible to compare the predicted and measured PET to verify their range.

### Depth Error Verification

#### Verification Result of an Irradiation Field

**Figure 1:**
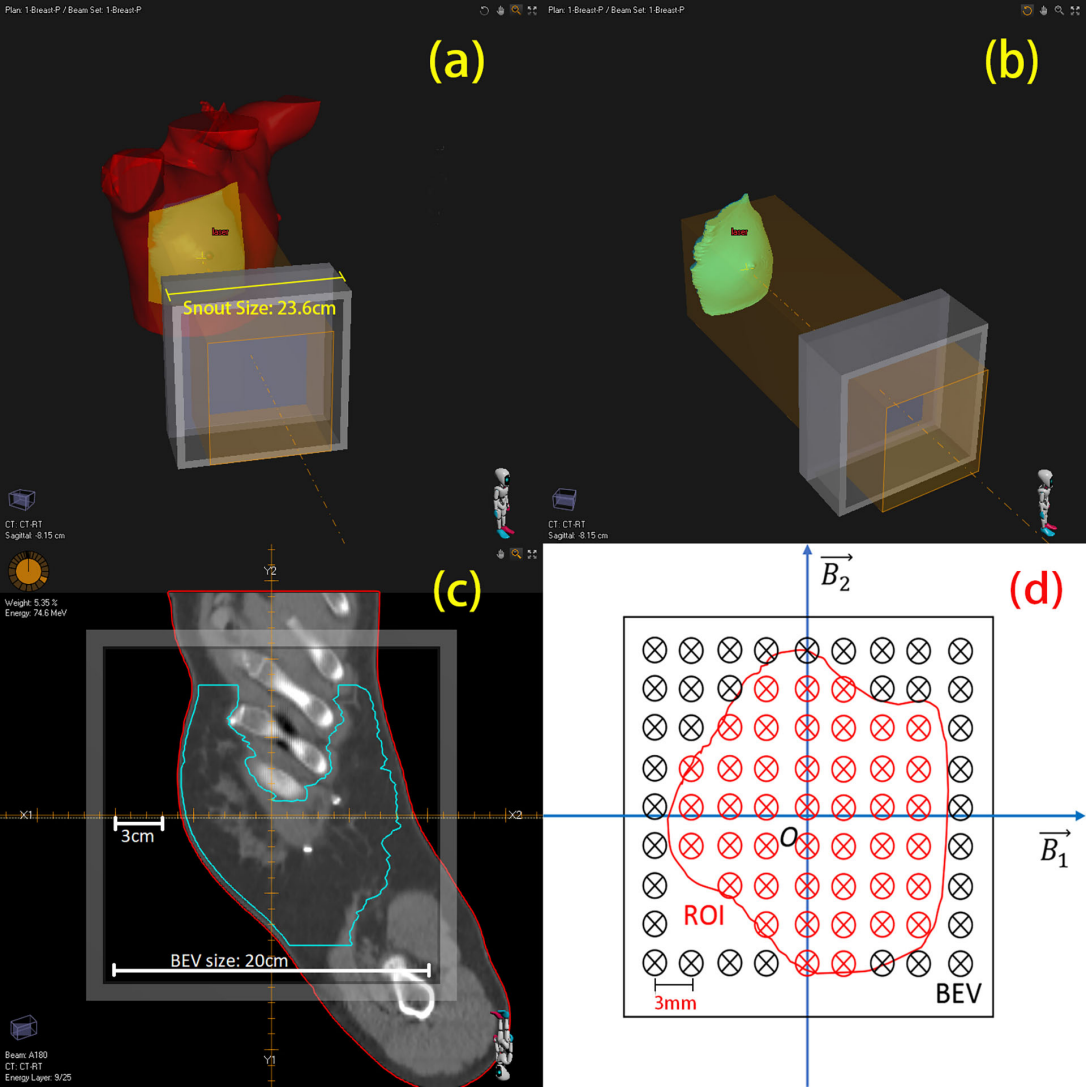
3-D view of region of interest (ROI) and mapping to BEV (beam’s eye view). **(A)** A single beam irradiates to the patient’s breast. **(B)** Irradiating to the patient CTV. **(C)** One slice image of CTV on BEV. **(D)** Sketch of scoring lines inside (red) and outside (black) the ROI; scoring line interval is 3 mm.

In each treatment plan, there could be one or more irradiation fields to provide a sufficient dose covering to the tumor tissue. In the treatment, the planned dose is delivered to the tumor tissue marked in “region of interest (ROI)”, which covers a certain 3-D region of the patient body (**Figures 1A, B**). One-dimensional PET scoring lines are drawn along the beam direction perpendicular to the beam’s eye view (BEV) plane (10). From the BEV, as shown in **Figure 1C**, the PET curve scoring line is uniformly distributed with an identical fixed interval (3 mm in our cases).

Obviously, not all the data from the scoring lines are useful to us. As the CTV is of our concern, we project this 3-D ROI on the BEV plane as a 2-D area, which circled a group of scoring lines (**Figure 1D**, just a sketch not real case). Only the scoring lines inside the ROI will be involved to calculate the mean value and standard deviation of the depth error of an irradiation field. This method of observing from the beam sight has been reported in many studies (6, 20–22). The comparison between two 3D-PET images is then degraded to one-dimensional curve problems; the statistical result of a case is then generated from the collection of one-dimensional curve comparison results.

#### *R*_50_ Method: Point-Based

**Figure 2:**
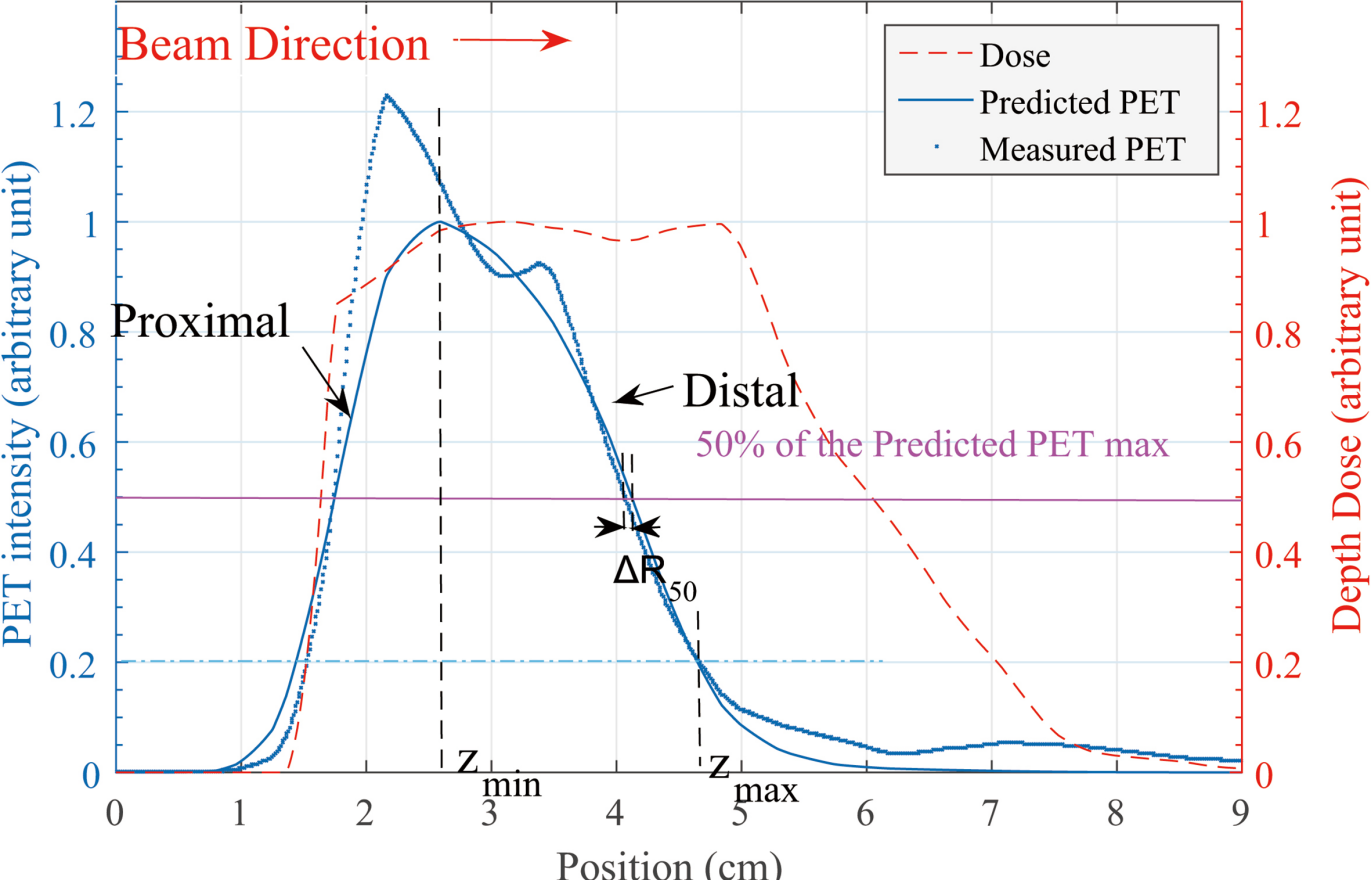
Predicted and measured PET 1-D curve. Along the beam direction, the depth error at distal edge is our concern. Definition of Δ*R*_50_ is shown in this figure. *z_min_* and *z_max_* location of the *R_shift_* method is defined on the predicted PET curve.

In our verification cases, the predicted and measured PET curves had similar shapes and edges, but the distal edges did not have the same position and slope. *R*_50_ is defined as the position where the PET intensity is 50% of its maximum (3). The predicted PET curve is set as a reference, and a horizontal marking line is drawn at half of the maximum level. The horizontal line marked out two points at the distal side of the curves while the depth error calculated based on *R*_50_ is defined as Δ*R*_50_ = *R*_50,_ *_pred_* - *R*_50_ *_,meas_*. This process is shown in **Figure 2**.

#### *R_shift_* Method: Segment-Based

Contrary to the *R*_50_ method, if we are not concerned about a certain point but a segment of the curve, then the depth error evaluation reflects the feature of the entire segment rather than a single point. One method to calculate the depth error for a segment of a curve is the *R_shift_* method (3, 10, 20).

The kernel of the shift method is to find a shift distance *δ* that minimizes the difference between predicted and measured PET curves [Eq. (1)], this difference is presented as a cost function *f*(*δ*) [Eq. (2)].


(1) $$\Delta R_{shift}=\delta_{0}\;\text{where}\;f(\delta_{0})=\min\{f(\delta )\}$$

(2) $$f(\delta )=\{\begin{array}{l} \int_{z_{\min}}^{z_{\max}}\left|A_{pred}(Z)-A_{meas}(Z-\delta )\right|dz,\;\mathrm{continuous}\\ \sum_{i=0}^{M}\left|A_{pred}(Z_{i})-A_{meas}(Z_{i}-\delta )\right|,\;\mathrm{discrete} \end{array}$$

In Eq. (2), A(z) is the PET activity depth distribution along the beam direction (for prediction and measurement, respectively) and *δ* is specified as a depth shift between two curves. The reason of the entire curve deviation between predicted and measured PET activity data is not fully understood in this work; by specifying the integral region [Z_min_ and Z*_max_* in Eq. (2)], we focus on the curve difference in the distal edge area rather than the entire curve. We specify the Z*_min_* as the peak position of the predicted PET curve and the Z*_max_* as 20% of the maximum location (**Figure 2**).

**Figure 3:**
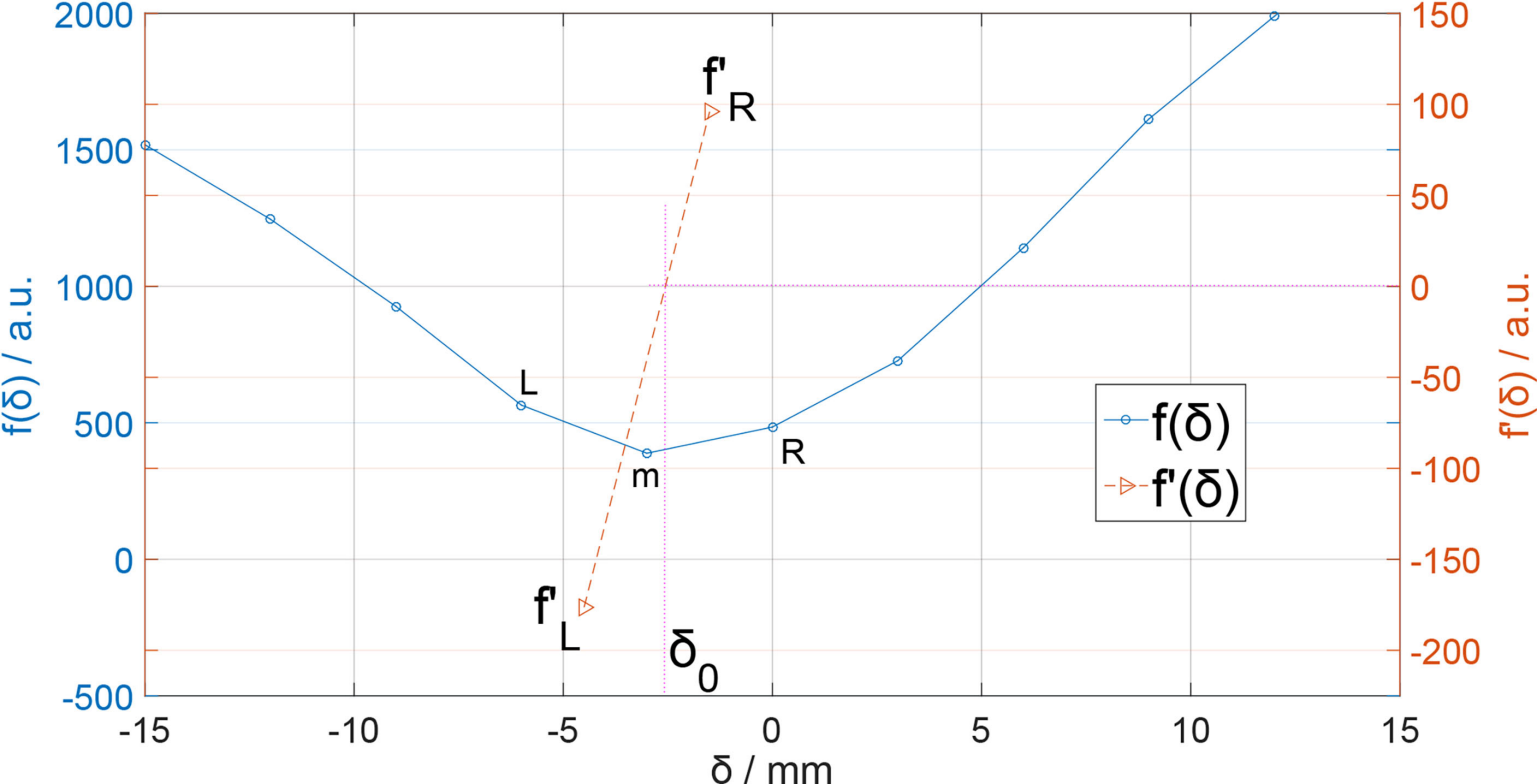
*f*(*δ*) and *f'*(*δ*) for seeking *δ*_0_. The position of *δ*_0_ is located where *f'*(*δ*_0_) = 0.

In Eq. (2), the cost function *f*(*δ*) describes the difference between continuous curves in a region, but the data stored in the computer are always discrete data lists. Therefore, cost function *f*(*δ*) has both continuous and discrete descriptions. Here, in the discrete description of Eq. (2), *i* ∈ [0, *M*] is a list subscript index corresponding to the region [Z_min_, Z_max_] presented as a discretization data list *Z_i_*, and the value of *δ* should be chosen discretely with the same value interval of *Z_i_*, which is 3 mm, the same as our data grid voxel size and the curve’s spatial resolution. The value of *δ* is set in [–15,15]mm with a range step of 3 mm and *f*(*δ*) is plotted in **Figure 3**.

Obviously, there is a minimum value point on the *f*(*δ*) curve. If our *δ* sampling density was high enough, we can easily locate the minimum point of the *f*(*δ*) curve and the corresponding *δ* would be the Δ*R* for curves. In our case, however, the value of *δ* sampling step was 3 mm, which is not small enough. Here, we can use derivative interpolation to accurately find the minimum point of the *f*(*δ*) curve. In *f*(*δ*) discrete list, there is a minimum *f*(*δ_m_*) with corresponding *δ_m_*, which is in the discrete *δ* list (**Figure 3**); the point is recorded as point *m*[*δ_m_*, *f*(*δ_m_*)]. On the left and right side of point *m*, there are point *L*[*δ_m-1_*, *f*(*δ_m-1_*)] and point *R*[*δ_m+1_*, *f*(*δ_m+1_*)]. Obviously, the minimum point [*δ*_0_,*f*(*δ*_0_)] of *f*(*δ*) is between L and R. Then, we calculate the left and right derivative of point *m*:


(3) $$f_{L}^{'}=\frac{f(\delta_{m})-f(\delta_{m-1})}{\delta_{m}-\delta_{m-1}}\text{ and }f_{R}^{'}=\frac{f(\delta_{m+1})-f(\delta_{m})}{\delta_{m+1}-\delta_{m}}$$

We know that *f*(*δ_m_*) is smaller than *f*(*δ_m-1_*) and *f*(*δ_m+1_*), so $f_{L}^{'}<0\;and\;f_{R}^{'}>0$ ; therefore, there must be a *f'*(*δ*_0_) = 0 and the corresponding *δ*_0_ is exactly the Δ*R_shift_* we want. We can easily estimate the *δ*_0_ by linearly interpolating $f_{L}^{'}\;and\;f_{R}^{'}$ :


(4) $$\delta_{0}=\delta_{m}-\Delta \delta \left[\frac{f_{R}^{'}+f_{L}^{'}}{2(f_{R}^{'}+f_{L}^{'})}\right]$$

Here, Δ*δ* = 3mm is our data grid voxel size, and *δ_m_* can be searched from the discrete *f*(*δ*) list. The *δ*_0_ in Eq. (4) is the point where *f*(*δ*) is minimized; thus, the integration in Eq. (1) is minimized, so the *δ*_0_ here is the Δ*R_shift_* we want.

## Results

Both *R*_50_ and R*_shift_* methods are applied to analyze the depth error of two fields of each patient. Depending on different types of dose distribution, depth error is scored and calculated for CTV, boost area, or both. The statistical result is in **Table 1**.

**Table 1**. Enrolled breast cancer patient information: age, planning dose, field, time course, and all their depth error. All patients are two-field cases. Patients may have different delay times before positron emission tomography scanning, but the data acquisition (Acq) time is the same for all cases. The Δ*R*_50_ and Δ*R_shift_* are scored in the CTV region and boost region (if exist). Different dose distribution strategies: Uniform: a dose is uniformly distributed in the CTV region in each fraction; Boost: dose is delivered to the tumor bed region in one fraction; SIB (simultaneous integrated boost): uniform and boost dose are simultaneously delivered in one fraction.

The mean value and standard deviation in **Table 1** is calculated as:


(5) $$\text{mean}=\bar{x}=\frac{1}{n}\Sigma_{i=1}^{n}x_{i},\;\text{std}:\;\sigma =\frac{1}{n}\sqrt{\Sigma_{i=1}^{n}{\left(x_{i}-\bar{x}\right)}^{2}}$$

**Figure 4:**
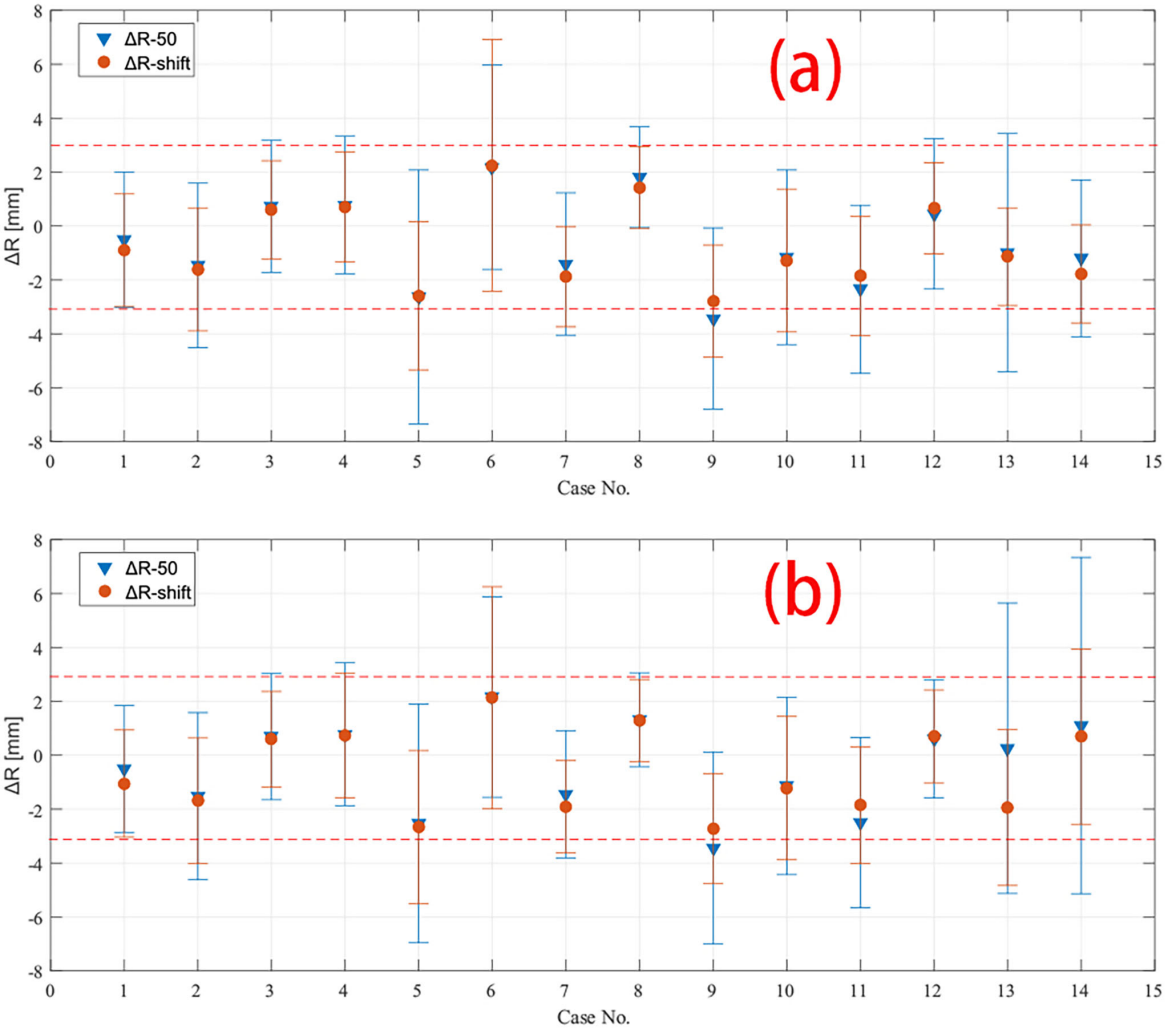
Depth error of all 14 cases calculated by both *R*_50_ and *R_shift_* methods in **(A)** field 1 and **(B)** field 2. The mean and standard deviation of depth error in CTV region of all cases are plotted except case 7 and 8, which have only data in boost region to be plotted. The red dashed line marked −3 mm and 3 mm in the plot.

The mean value of the depth error in all these 14 cases is within the range of [−3, 3] mm for both *R*_50_ and *R_shift_* methods, except case 9 CTV region with the *R*_50_ method. The mean value and standard deviation of depth error of all cases are plotted in **Figure 4**, where the predicted PET depth is deeper than the measured PET if the Δ*R* is a positive value and *vice versa*. We can see that most patients have similar mean depth error calculated by *R*_50_ and *R_shift_* methods in both field 1 and field 2. To evaluate whether the mean depth error from the two methods has significant difference, a paired Student’s *t*-test is applied. Setting the confidence level *α* = 0.01 and testing all the mean value of depth error, we get *p* = 0.541 > *α* which means the mean value of depth error has no significant difference between *R*_50_ and *R_shift_* methods. Moreover, we also find that all cases have a smaller standard deviation of depth error calculated by *R_shift_* rather than *R*_50_ except case 6. This can be roughly explained as the segment-based method refers more information to calculate a depth error result, which leads to higher robustness compared to *R*_50_, thus presenting a smaller standard deviation.

**Figure 5:**
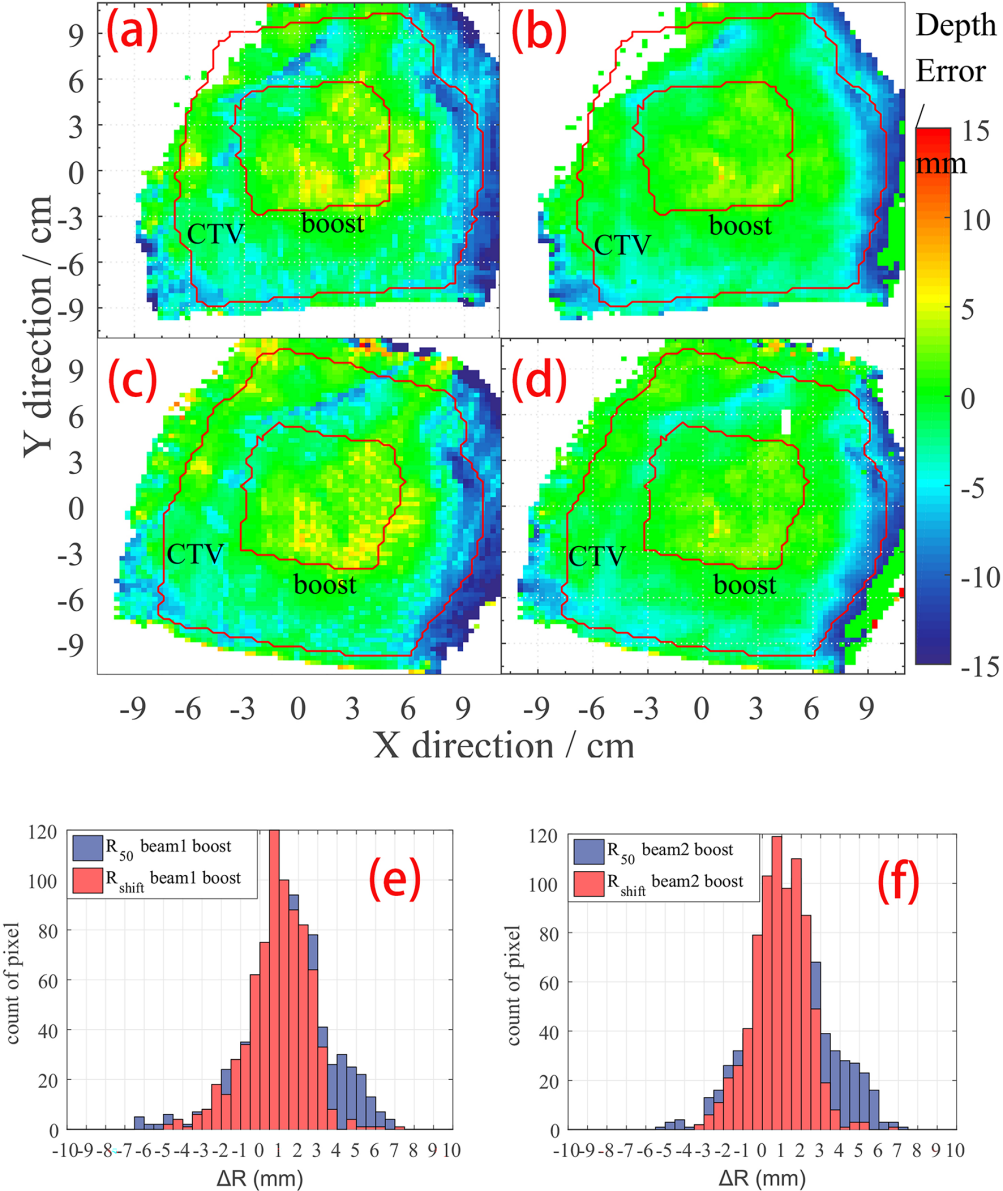
Δ*R* detail result of case no. 10. The CTV and boost areas are marked within the red curve. **(A)** *R*_50_ depth error map on beam 1; **(B)** *R_shift_* depth error map on beam 1; **(C)** *R*_50_ depth error map on beam 2; **(D)** *R_shift_* depth error map on beam 2. Histogram of depth error statistic on boost region on **(E)** beam 1 view, and **(F)** beam 2 view of case no. 10.

Besides a global result analysis in **Figure 4**, a detailed result of a case is shown in **Figure 5**. The CTV and boost ROI is projected on the BEV plane whose edge circles an area, as shown in **Figures 5A–D**. Inside the boost region, we can see several positive Δ*R* pixels, and in **Table 1**, we can see that the mean value of Δ*R* of case 10 in the boost region is a positive number. In addition, the result of the *R_shift_* method (**Figures 5B, D**) shows a smoother Δ*R* distribution than the *R*_50_ method (**Figures 5A, C**). The Δ*R* distribution in the boost region is also plotted as a histogram in **Figures 5E, F**, which indicates that *R*_50_ and *R_shift_* methods give similar mean depth errors but different distribution widths.

## Discussion

### *Z_min_* and *Z_max_* in *R_shift_* Integration

In our calculations with the *R_shift_* method (*Section R50 Method: Point-Based*), the integration range [*z_min_*, *z_max_*] in Eq. (1) is simply specified as the range from the peak position to 20% of the peak value on the distal tail of the curve. Herein, the dose delivery is limited within the breast area with a high carbon component and low biological washout rate. Therefore, the PET image shows a smooth single peak curve if we score the data along the beam direction. Under this condition, we do not need to define a complex method to find out a specific *z_min_* and *z_max_* for Eq. (1). Frey provides a method (20) to locate *z_min_* and *z_max_* that can optimize the curve shape of *f*(*δ*) to achieve a more reasonable depth error result. In our study, however, the anatomical structure is not complex, which provides good stability to the result, so that *z_min_* and *z_max_* need no further optimization.

### “Off-line” PET and “In-beam” PET

“Off-line” PET is applied in this work. The “off-line” PET scanner with a full ring detector system has been widely used in nuclear medicine clinical application. Mature device and reconstruction algorithm reduces the cost of use. However, the long transport time makes the contribution of short-lived isotopes undetectable; also, the co-registration between plan-CT and PET-CT may introduce additional geometrical error.

“In-beam” PET, on the other hand, can acquire the induced PET signal in time and thus collect the activity contributed by short-life isotopes. Higher signal intensity gives higher image accuracy and thus better comparison results in data analyzing. However, the in-beam PET requires a complex PET scanner installed inside the treatment room, and it is hard to construct a full ring to avoid blocking the beam path. A PET scanner with an incomplete ring will introduce more noise to data reconstruction and will need a more accurate reconstruction algorithm.

The breast cancer cases in this work have a relative higher yield of long-life isotope (like carbon-11); thus, “off-line” PET is applicable in this work.

## Conclusions

PET could be used in depth error verification after proton therapy. Point-based and segment-based methods show no significant difference in the mean depth error result, which is within [−3, 3] mm. The segment-based methods have higher robustness with smaller standard deviation, while the point-based method has a relatively larger standard deviation. Both point-based and segment-based methods are suitable in the data analysis of our breast cancer cases, while the point-based method is more appropriate because of the low cost in the calculation.

## Data Availability Statement

The original contributions presented in the study are included in the article/supplementary material. Further inquiries can be directed to the corresponding authors.

## Ethics Statement

The studies involving human participants were reviewed and approved by the Ethics Committee of Shanghai Proton and Heavy Ion Center. The patients/participants provided their written informed consent to participate in this study. Written informed consent was obtained from the individual(s) for the publication of any potentially identifiable images or data included in this article.

## Author Contributions

JZ was responsible for writing the original draft. YL was responsible for writing the original draft and review. YXS and WW were responsible for methodology. ZH was responsible for data curation. JC was responsible for conceptualization. RZ was responsible for supervision. YS was responsible for funding acquisition. All authors contributed to the article and approved the submitted version.

## Funding

This project was supported by the Shanghai Municipal Health Commission (Grant No. 202040279), the Pudong New Area Science and Technology Development Foundation (No. PKJ 2020-Y56), and the Natural Science Foundation of Shanghai (21ZR1460300).

## Conflict of Interest

The authors declare that the research was conducted in the absence of any commercial or financial relationships that could be construed as a potential conflict of interest.

The reviewer WH declared a past co-authorship with the authors to the handling editor.

## Publisher’s Note

All claims expressed in this article are solely those of the authors and do not necessarily represent those of their affiliated organizations, or those of the publisher, the editors and the reviewers. Any product that may be evaluated in this article, or claim that may be made by its manufacturer, is not guaranteed or endorsed by the publisher.
