# Corrigendum: A comparative study of two *in vivo* PET verification methods in clinical cases

**DOI:** 10.3389/fonc.2024.1507589

**Published:** 2024-11-12

**Authors:** Junyu Zhang, Yan Lu, Yinxiangzi Sheng, Weiwei Wang, Zhengshan Hong, Yun Sun, Rong Zhou, Jingyi Cheng

**Affiliations:** ^1^ Department of Nuclear Medicine, Shanghai Proton and Heavy Ion Center, Fudan University Cancer Hospital, Shanghai, China; ^2^ Shanghai Key Laboratory of Radiation Oncology, Shanghai, China; ^3^ Shanghai Engineering Research Center of Proton and Heavy Ion Radiation Therapy, Shanghai, China; ^4^ College of Physics, Sichuan University, Chengdu, China; ^5^ Key Laboratory of Radiation Physics and Technology, Ministry of Education, Chengdu, China; ^6^ Department of Medical Physics, Shanghai Proton and Heavy Ion Center, Shanghai, China; ^7^ Department of Radiotherapy, Shanghai Proton and Heavy Ion Center, Shanghai, China

**Keywords:** proton therapy, breast cancer, positron emission tomography, depth verification, methods comparison

In the published article, there was an error in the **Funding** statement. The funding statement for the Natural Science Foundation of Shanghai was displayed as “21ZR460300”. The correct statement is “Natural Science Foundation of Shanghai (21ZR1460300)’’.

The correct **Funding** statement appears below.

“This project was supported by the Shanghai Municipal Health Commission (Grant No. 202040279), the Pudong New Area Science and Technology Development Foundation (No. PKJ 2020-Y56), and the Natural Science Foundation of Shanghai (21ZR1460300).’’

The authors apologize for this error and state that this does not change the scientific conclusions of the article in any way. The original article has been updated.

